# Place des thèses et mémoires dans les publications scientifiques des enseignants de dermato-vénéréologie de l’université de Lomé (Togo): Ce travail a fait l’objet d’une communication orale lors des 5^e^ journées dermatologiques de Ouagadougou (Burkina Faso) en octobre 2017

**DOI:** 10.48327/V6RZ-NP67

**Published:** 2021-02-18

**Authors:** G. Mahamadou, B. Saka, W. Gnassingbé, S. Akakpo, J. Téclessou, K. Kombaté, K. Tchangai-Walla, P. Pitché

**Affiliations:** 1 Service de Dermatologie et IST, CHU Sylvanus Olympio, Université de Lomé, Togo; 2 Service de Dermatologie et IST, CHU Campus, Université de Lomé, Togo

**Keywords:** Thèses/mémoires, Publication, Dermato-vénéréologie, Togo, Theses/dissertations, Publication, Dermatology, venereology, Togo

## Abstract

L’objectif de cette étude était de situer la place des thèses et mémoires (de techniciens supérieurs de santé, de D.E.S ou de master) dans les publications scientifiques des enseignants de dermato-vénéréologie de l’Université de Lomé (Togo). Nous avons recensé les thèses et mémoires ayant porté sur la dermato-vénéréologie entre 1990 et 2016, dans trois établissements de l’Université de Lomé et consulté des bases de données (Medline, Inist, registres du service) pour rechercher les publications des enseignants durant cette période. Au total 41 thèses et 50 mémoires ont été réalisés sur les dermatoses infectieuses et les IST/VIH (46,1 %), les dermatoses immunoallergiques (11,0 %) et les dermatoses tumorales (8,8 %). Sur ces 91 travaux, 56 (dont 28 thèses) ont été publiés, dans des revues indexées (21 thèses et 26 mémoires) ou non indexées (7 thèses et 2 mémoires). Ces 56 publications représentaient 27,7 % des 202 publications faites par les enseignants de dermato-vénéréologie de l’université de Lomé. Sur les 28 thèses publiées, le thésard était le premier auteur dans un cas (3,6 %). Ce travail montre que les thèses et mémoires de dermato-vénéréologie représentent presqu’un tiers des publications de cette discipline au Togo.

## Abstract (en)

The aim of this study was to situate the place of theses and dissertations (senior healthcare technicians, D.E.S or master) in the scientific publications of dermato-venerology teachers at the University of Lomé (Togo). We listed the theses and dissertations on dermato-venerology between 1990 and 2016, in three institutions of the University of Lomé and consulted databases (Medline, Inist, registers of the service) to search for publications by teachers during this period. A total of 41 theses and 50 dissertations were completed, on infectious dermatoses and STI/HIV (46.1%), immunoallergic dermatoses (11.0%) and tumour dermatoses (8.8%). Of these 91 works, 56 (including 28 theses) were published in indexed (21 theses and 26 dissertations) or non-indexed (7 theses and 2 dissertations) journals. These 56 publications represented 27.7% of the 202 publications made by the dermato-venerology teachers at the University of Lomé during this period. Of the 28 published theses, the MD student was the first author in one case (3,6%) This work shows that theses and dissertations in dermato-venerology represent almost one third of the publications in this discipline in Togo.

## Introduction

La thèse pour le diplôme d’état de docteur en médecine (ou en pharmacie) et le mémoire sont des dissertations scientifiques portant sur un travail de recherche conduit par un étudiant, qui marquent la fin des études ou d’un cycle universitaire [1]. Pour l’obtention de licences, masters, doctorats ou de diplômes d’études spécialisées, beaucoup d’étudiants inscrits dans l’un de ces parcours académiques de formation des agents de santé, mènent des travaux de recherche en dermato-vénéréologie avec l’encadrement d’un enseignant-chercheur. Ces travaux sont diffusés à travers deux principaux moyens, les publications et les communications au cours des réunions ou congrès scientifiques. Mais, en pratique, très peu de travaux de thèses de médecine font l’objet de publications [2]. En effet, des travaux antérieurs ont montré qu’à l’Université de Lomé (Togo), la valeur scientifique des thèses de médecine restait hétérogène et une faible proportion était valorisée en publication scientifique ou communication [1,2]. Ces travaux concernaient les thèses de médecine générale [2] et les mémoires de pédiatres [1], mais aucun n’a concerné la dermato-vénéréologie. Cette étude situe l’importance des thèses/mémoires dans la production scientifique des enseignants de dermato-vénéréologie de l’université de Lomé (Togo).

## MatÉriels et mÉthodes

Une étude transversale a été menée sur les thèses et mémoires portant sur la dermato-vénéréologie et soutenus à la faculté des sciences de la santé, à l’école des assistants médicaux et à l’institut national des sciences de l’éducation de l’université de Lomé de 1990 à 2016. Durant cette période, le nombre des enseignants chercheurs de dermato-vénéréologie de cette université, est passé de trois entre 1990-1999 à sept entre 2010 et 2016. La collecte des données a été effectuée à l’aide d’une grille de collecte préétablie i) à la bibliothèque des services de dermato-vénéréologie des CHU Sylvanus OLYMPIO, Campus, et Kara où exercent les dermatologues hospitalo-universitaires du pays; ii) dans les registres de soutenance de thèses et/ou mémoires des trois établissements de l’Université de Lomé (école des assistants médicaux et à l’institut national des sciences de l’éducation de l’université de Lomé); et iii) par la consultation de deux bases de données que sont *Medline/Pubmed* et CNRS-Inist/Pascal. Nous avons considéré ces deux bases de données à cause de leur poids dans les différentes publications scientifiques.

Nous avons donc relevé dans ces trois registres, les titres des thèses et mémoires soutenus avec les noms des candidats, des directeurs et codirecteurs dermato-vénérologues. Ensuite, nous avons organisé une entrevue avec les directeurs et codirecteurs de ces thèses/mémoires afin de déterminer ceux qui ont été diffusés (publications et/ou communications). Enfin, la consultation des bases de données *Medline/Pubmed* et CNRS-Inist/Pascal a permis de déterminer les articles issus des thèses/mémoires parus dans des revues indexées ou non. Cette recherche a été effectuée à partir des noms des candidats et du directeur de thèse/mémoires utilisés comme des mots-clés. Tous les articles parus dans les revues référencées par *Medline/Pubmed* et CNRS-Inist/Pascal ont été considérés comme publications indexées. Les articles non retrouvés dans ces deux bases de données, mais publiés dans des revues nationales et régionales ont été considérés comme travaux non indexés. Une revue a été qualifié de nationale quand elle est éditée au Togo, régionale quand elle est éditée en Afrique et internationale lorsqu’elle est éditée hors du continent Africain.

Ces articles ont été identifiés à partir des différents dossiers des enseignants chercheurs aux différentes épreuves du conseil africain et malgache de l’enseignement supérieur (CAMES). La consultation des bases de données *Medline/Pubmed* et CNRS-Inist/Pascal a permis de déterminer le nombre total des articles publiés dans des revues indexées par les enseignants de l’équipe de dermato-vénéréologie au cours de la période d’étude. Pour déterminer ce nombre, nous avons préalablement introduit dans l’interface de recherche de ces deux bases de données les noms des enseignants. Ensuite, nous avons dénombré tous les articles par enseignant. Afin d’éviter de compter deux fois un même article, nous avons tenu compte de la position d’auteur (première, deuxième ou dernière position) et du thème de recherche pour attribuer la paternité (auteur ou co-auteur) d’un travail à un enseignant.

## RÉsultats

Durant la période d’étude, 91 travaux (dont 41 thèses) ont porté sur la dermato-vénéréologie, soit 19 (20,9 %) entre 1990-1999 versus 45 (49,5 %) entre 2010-2016. En se rapportant au nombre d’enseignants, les ratios thèse/enseignant sur la période totale et thèse/enseignant annuel étaient de 13,6 et de 0,5 respectivement. Les ratios mémoire/enseignant sur la période totale et mémoire/enseignant annuel étaient respectivement de 10 et de 0,37. La moitié des travaux était des études rétrospectives (51,6 %) suivie des études prospectives et/ou transversales (30,8 %), des cas et séries de cas cliniques (9,9 %), des études cas-témoins (3,3 %), des enquêtes CAP (2,2 %), une revue de littérature (1,1 %) et un essai randomisé (1,1 %). Les pathologies abordées étaient les dermatoses infectieuses et IST/VIH (46,1 %), dermatoses immunoallergiques (11,0 %), panoramas de dermatoses (11,0 %), dermatoses tumorales (8,8 %), dermatoses inflammatoires/Connectivites (7,7 %). Les autres thèmes (cosmétologie, génodermatoses, rédaction médicale) ont représenté 9,9 % des travaux. Les aspects étudiés étaient d’ordre épidémiologique et clinique (38,5 %), épidémiologique clinique et thérapeutique (24,1 %), santé publique (20,9 %), clinique et/ou thérapeutique (5,5 %), épidémiologique et/ou thérapeutique (6,6 %), pharmacovigilance (3,3 %) et pédagogie (1,1 %). Sur les 91 travaux, 56 (61,5 %) dont 28 thèses ont été publiés dans des revues scientifiques. Parmi elles, 47 (51,6 %) l’ont été dans des revues indexées (dont 21 thèses) et neuf (dont sept thèses) dans des revues non indexées. Il s’agissait de 78,6 % de revues internationales, 16,1 % de revues régionales (Tableau I). Les revues: i) *Médecine et Santé Tropicales,* ii) *Annales de Dermatologie et de Vénéréologie* et iii) *Bulletin de la Société de Pathologie Exotique* ont été des revues internationales choisies dans plus de la moitié des articles indexés (Tableau I). Le taux de publication était de 58 % entre 1990-1999, 63 % entre 2000-2009 et 62 % entre 2010-2016. Sur les 28 thèses publiées, le thésard était le premier auteur dans un cas (3,6 %) et dans une revue indexée. Par ailleurs, 50 (55 %) de ces travaux ont été diffusés au cours de 63 communications lors des rencontres scientifiques, certains travaux pouvant être communiqués plusieurs fois.

**Tableau I:** Articles dérivés des thèses/mémoires selon le type de revue Articles derived from thesis/dissertations according to the type of journal

		Nombre d’articles
	Revues indexées (47)	
**Revues internationales (44-78,6 %)**	*Médecine Tropicale/Médecine et Santé Tropicales*	12

	*Annales de Dermatologie et de Vénéréologie*	9

	*Bulletin de la Société de Pathologie Exotique*	6

	*Cahiers Santé*	4

	*Nouvelles Dermatologiques*	4

	*International Journal of Dermatology*	2

	*BMC Public Health*	2

	*Annales de Chirurgie Plastique Esthétique*	1

	*BMC Research Notes*	1

	*Plos One*	1

	*Revue de Médecine Interne*	1

	*ISRN Dermatology*	1

**Revues régionales (09-16,1 %)**	*Pan African Medical Journal*	3

	**Revues non indexées (9)**	

	*Médecine d’Afrique Noire*	3

	*Écho de Santé*	2

	*Guinée Médicale*	1

**Revues nationales (03-5,3 %)**	*Journal de la Recherche Scientifique de l’Université de Lomé*	3

	**Total**	**56**

Durant la même période, les enseignants de dermato-vénéréologie ont été auteurs et/ou co-auteurs de 202 publications dont 56 (27,7 %) issues des thèses et mémoires. Ces publications ont été faites dans des revues internationales (55,4 %), régionales (39,1 %) et nationales (5,5 %).

## Discussion

Ce travail évaluant la place des thèses et mémoires de dermato-vénéréologie dans la production scientifique de cette spécialité au Togo a montré que sur 91 travaux, 56 (dont 28 thèses) ont été publiés, dans des revues indexées (21 thèses et 26 mémoires) ou non indexées (7 thèses et 2 mémoires). Ces 56 publications représentaient 27,7 % des 202 publications faites par les enseignants de dermato-vénéréologie de l’université de Lomé

Entre 1990 et 1999, le nombre des thèses/mémoires soutenus était de 19 (20,9 %) contre 45 (49,5 %) pour la période de 2010 à 2016. On s’aperçoit que l’effectif des travaux a plus que doublé entre les deux périodes. Cet accroissement pourrait s’expliquer surtout par l’augmentation du nombre des enseignants de dermato-vénéréologie passant de trois entre 1990-1999 à sept entre 2010-2016. Dans cette étude, la moitié des travaux portait sur des séries rétrospectives (51,6 %) suivie des séries prospectives et/ou transversales. Cette forte proportion de séries rétrospectives pourrait s’expliquer par la facilité de réalisation de ces types d’études, l’absence de financement pour la recherche. Mais, l’analyse des séries rétrospectives est limitée par le manque de certaines données et le nombre élevé de dossiers médicaux inexploitables [3].

Les dermatoses infectieuses et IST/VIH/SIDA étaient les principaux thèmes abordés par les travaux avec une proportion de 46,1 %, ceci au regard du contexte épidémiologique au Togo caractérisé par une forte prévalence des dermatoses infectieuses. La pandémie du VIH a changé l’expression clinique de plusieurs pathologies dermatologiques [4,5] expliquant ainsi le nombre élevé de travaux dédiés aux IST/VIH/SIDA. Cette prédominance des pathologies infectieuses a été retrouvée par Gbadoe et al [1] dans 101 thèses et mémoires en pédiatrie au Togo.

Les sujets abordés au cours des travaux scientifiques étaient essentiellement d’ordre épidémiologique et clinique dans 38,5 % des cas. Ce résultat est comparable à celui de Pitché et al [2] en 2007 dans l’étude se rapportant à l’ensemble des thèses soutenues à la Faculté des Sciences de la Santé du Togo. La prédominance de ces deux catégories s’expliquerait par la facilité de mise en œuvre, mais aussi leur intérêt dans la recherche des facteurs étiologiques et démographiques de la plupart des grandes endémies et pathologies dans un pays tropical sous médicalisé. Par contre, les études de santé publique représentaient 20,9 %. Cela pourrait s’expliquer par le fait que ces études nécessitent des échantillons plus importants et représentatifs de la population générale avec un coût financier élevé alors que la plupart des travaux ont été réalisés en milieu hospitalier.

La proportion de thèses et mémoires publiés était de 61,5 % dont 68,3 % de thèses (28/41). Parmi ces publications, 51,6 % (21 thèses et 26 mémoires) ont été faites dans des revues indexées correspondant à 51,2 % des thèses et 52 % des mémoires. La forte proportion de publications dans les revues indexées a une très grande valeur d’évaluation interne des travaux scientifiques réalisés par les enseignants de dermato-vénéréologie, même si la plupart de ces revues indexées ont des facteurs d’impact presque nul, ou qu’elles sont *open access* ou prédatrices. Ces ratios de publication sont supérieurs aux autres séries dans la littérature [2,6-11] (Tableau II). En effet, Pitché et al [2] ont trouvé 41 % de thèses de médecine publiées contre 68,3 % de thèses de dermato-vénéréologie dans cette étude. Nos données représentaient plus du double si on prend en compte les thèses publiées dans les revues indexées (51,2 % vs 22,5 %). Les revues *Médecine et Santé Tropicales, Annales de Dermatologie et de Vénéréologie* et *Bulletin de la Société de Pathologie Exotique* ont été les périodiques sollicités dans plus de la moitié des articles indexés. Cette forte proportion serait liée à l’accessibilité linguistique et financière, car ce sont des revues paraissant en Français, mais aussi le fort taux d’acceptation de certaines d’entre elles. Le souci de visibilité des auteurs, en publiant dans des revues indexées a été un fondement majeur de ce choix, même si ces revues françaises ont un facteur d’impact relativement faible. Ces résultats témoignent de la valorisation des thèses et mémoires initiés par les enseignants de dermato-vénéréologie, car bien que la qualité de la thèse influence sa publication [7], la motivation de l’enseignant et sa maîtrise des règles rédactionnelles constituent des atouts importants. Les publications issues des thèses et mémoires ne représentent que 27,7 % des publications des enseignants de dermato-vénéréologie, avec une très faible proportion d’articles (3,6 %) signée par le thésard. En effet, presque toutes ces publications ont été signées par le directeur de thèse ou un autre enseignant de dermatologie en quête d’une promotion sur le plan universitaire, les publications étant un critère majeur. Il ne s’agit nullement d’un problème d’intérêt scientifique qu’accordent les enseignants de dermato-vénéréologie de l’université de Lomé aux thèses/mémoires. A titre illustratif à Monastir (Tunisie) entre 2001-2005, le ratio thèse/enseignant était de 3,25 et le ratio thèse-enseignant annuel était de 0,65 dans le service dermatologie [9], alors qu’ils étaient respectivement de 13,6 et 0,5 au Togo. La production scientifique permet d’améliorer la qualité des soins et celle de l’enseignement, c’est pourquoi plusieurs publications ont été faites en dehors des thèses/mémoires.

**Tableau II:** Comparaison des taux de publication de thèses en fonction des études Comparison of the publication rates of theses according to the type of studies

	Université	% thèses publiées: revues indexées/toutes	Période
Notre étude	Lomé (Togo)	51,2/68,3	1990-2016

Pitché et al [2]	Lomé (Togo)*	22,5/41	1993-2002

Balva et al [6]	Angers (France)	np/14	2007-2009

Baufreton et al [7]	Angers (France)	np/28	2002-2008

Benotmane et al [8]	Lille (France)	np/11,3	2001-2007

Ben Salem et al [9]	Monastir (Tunisie)	np/14,3	2001-2005

Carpentier et al. [10]	Brest (France)	np/13,2	2001-2005

Foucheyrand [11]	Tours (France)	np/30,3	1989-1992

Notes: travail portant sur les thèses (toutes spécialités confondues) de la faculté de médecine np: pourcentage non précisé dans l’article cité en référence

## Limites

La détermination du nombre d’articles des enseignants a été réalisée par la lecture des titres et résumés. Certains titres de thèses/mémoires étaient peu explicites de la réelle catégorie du travail. La principale limite de cette étude réside dans la question de l’indexation des revues scientifiques car certaines revues dans ces bases de données sont prédatrices. De même, des revues non indexées peuvent ne pas être des revues sérieuses avec une révision par les pairs, la comparaison est délicate. Même si les enseignants de dermatologie de l’université de Lomé privilégient les revues à comité de lecture, critère essentiel du CAMES pour la validation des articles pour les dossiers universitaires. Par ailleurs, ces enseignants évitent systématiquement les revues adressant des courriers pour solliciter les publications et celles demandant des quotes-parts pour être publié.

## Conclusion

Ce travail a mis évidence un taux de publication élevé des thèses et mémoires en dermato-vénéréologie à Lomé. Cependant, la part de ces thèses et mémoires dans la production scientifique des enseignants de dermato-vénéréologie était faible. Il a également permis d’apprécier l’apport de la thèse et du mémoire dans l’activité scientifique du service de Dermato-vénéréologie et la contribution de ces travaux dans le rayonnement du service, lequel dépend de la qualité des soins, de l’enseignement et des publications qui en sont issues.

## Remerciements

Au Doyen de la Faculté des Sciences de la Santé (FSS/UL) et aux Directeurs de l’Ecole des Assistants Médicaux (EAM) et de l’Institut National des Sciences de l’Education (INSE) qui ont autorisé l’accès aux bibliothèques de leurs établissements.

## Conflits D'intérêts

Les auteurs ne déclarent aucun conflit d’intérêts.
